# Selective Arterial Catheterization as a Bridging Therapy for Blunt Traumatic Aortic Injury With Intraperitoneal Organ Malperfusion

**DOI:** 10.7759/cureus.49060

**Published:** 2023-11-19

**Authors:** Koji Miura, Seigo Urushidani

**Affiliations:** 1 Emergency Medicine, Kurashiki Central Hospital, Kurashiki, JPN

**Keywords:** blunt aortic injury, blunt vascular trauma, malperfusion, thoracic endovascular aortic repair, aortic dissection, aortic injury, trauma, blunt thoracic aortic injury

## Abstract

Blunt thoracic aortic injury (BTAI) is fatal and requires thoracic endovascular aortic repair (TEVAR) for its optimal management. Performing TEVAR requires multidisciplinary specialists and supportive facilities.

We report a case of an 89-year-old man who presented to the emergency department with blunt trauma. Whole-body computed tomography (CT) revealed grade II aortic injury with disrupted blood flow to the left kidney. Sudden paralysis of the left lower extremity and distal progression of the aortic dissection occurred. However, TEVAR could not be performed immediately. Therefore, an external shunt from the right common femoral artery to the left lower extremity was created with angioplasty, superior mesenteric artery (SMA) stenting, and celiac artery (CA) balloon dilatation. The patient’s condition stabilized, and he was transferred to a hospital where TEVAR was performed.

Selective arterial catheterization (SAC) for treating intraperitoneal organ malperfusion caused by BTAI may be an effective bridging therapy for TEVAR.

## Introduction

Blunt thoracic aortic injury (BTAI) is a life-threatening condition. Most BTAI occurs in the arterial ligament attachment and aortic root due to deceleration forces, which result in aortic tears. BTAI is often complicated by polytraumas and is associated with a high risk of bleeding [1,2]. Malperfusion occurs in approximately 10% of non-traumatic type B aortic dissection cases [3]. However, cases of BTAI with malperfusion have rarely been reported, and their incidence is unknown. 

Thoracic endovascular aortic repair (TEVAR) has become the mainstay treatment for BTAI [1,2]. However, not all hospitals are equipped to perform TEVAR. Moreover, open surgery for polytraumas is challenging because of its complexity and invasiveness. Thus, some physicians may have no choice but to transfer patients whose general condition is unstable due to organ ischemia. Herein, we present the successful management of a case of BTAI with organ malperfusion using selective arterial catheterization (SAC), in a setting where immediate TEVAR was not feasible.

## Case presentation

An 89-year-old man with blunt trauma presented to the emergency department after a head-on collision with an onward approaching car. The patient complained of left lower abdominal pain. His vital signs were within normal limits. On examination, a seatbelt mark was observed on the right anterior iliac crest region. Immediate whole-body computed tomography (CT) revealed a left transverse process fracture of the first to third lumbar vertebrae, a hematoma in the left retroperitoneal zone II, and a grade II aortic injury from the tethered site of the arterial ligament to the left renal artery, resulting in the disruption of blood flow to the left kidney. Contrast-enhanced CT revealed the progression of the dissected lumen distal to the aortic bifurcation in the venous phase. 

**Figure 1:**
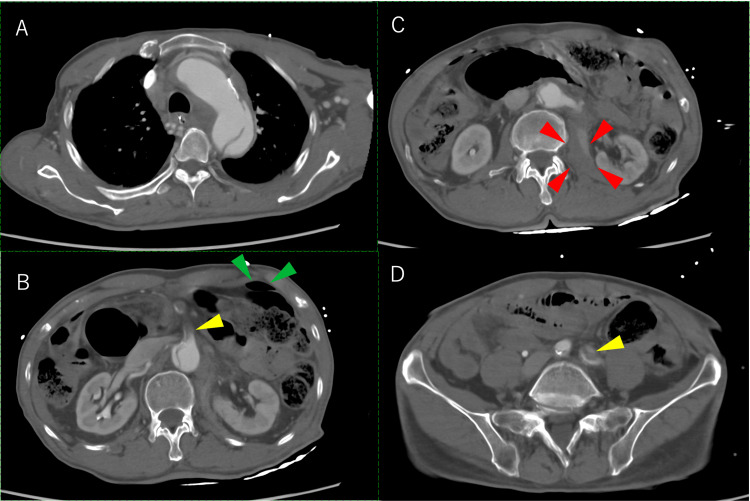
Contrast-enhanced CT after intubation (A) Contrast-enhanced CT after intubation showing aortic dissection and disruption of blood flow to the CA, SMA (B, yellow arrowheads), and left common iliac artery (C, red arrowheads). Free air was observed in the abdominal cavity (B, green arrowheads) and retroperitoneal hematoma (D, yellow arrowheads). CA, celiac artery; CT, computed tomography; SMA, superior mesenteric artery

The patient complained of sudden paralysis of the left lower extremity 72 minutes after the first CT, during intubation preparation for mild agitation. Repeated contrast-enhanced CT showed that the aortic dissection had progressed distally. Blood flow to the left common iliac artery, celiac artery (CA), and superior mesenteric artery (SMA) was disrupted, and the intestinal blood flow was extensively ischemic (Figure 1).

Though TEVAR was considered the best treatment, our hospital was not equipped to perform the procedure. Moreover, a nearby hospital that could perform TEVAR was unable to admit the patient immediately. Open surgery for aortic repair was deemed risky because of the patient’s advanced age and history of abdominal surgery for gastric and rectal cancers. Therefore, we performed an external shunt to the left lower extremity and immediate SAC for the SMA and CA to restore intraperitoneal organ perfusion. An 8-Fr sheath was placed cephalad from the right common femoral artery and caudally from the left common femoral artery in an ultrasound-guided manner. Immediately after reperfusion, the color tone of the left lower extremity improved. 

**Figure 2:**
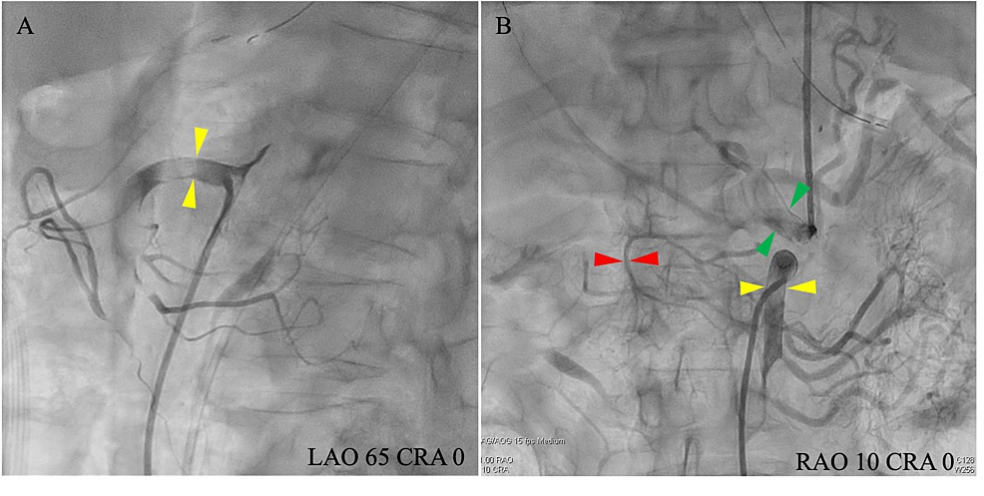
Selective angiography The selective angiography of the SMA revealed a highly stenotic true lumen (A, yellow arrowheads). Final selective angiography of the SMA (B, yellow arrowheads) and CA (B, green arrowheads) after stent placement and balloon dilation. Collateral blood flow from the SMA to the CA (B, red arrowheads) is shown. SMA, superior mesenteric artery; CA, celiac artery

Selective angiography for the SMA revealed a highly stenotic lumen. A stent was implanted from the root of the SMA to the periphery, and balloon dilation was performed. Similarly, balloon dilatation was done for the CA, and angiography confirmed improved blood flow from the CA and SMA (Figure 2).

Finally, an exploratory laparotomy was performed to establish good intestinal blood flow without necrosis. We repaired the small intestinal ruptures of 80 cm, 90 cm, and 120 cm from the ligament of Treitz. The left retroperitoneal zone II hematoma showed no enlargement. The abdomen was closed after thorough irrigation with saline solution.

**Figure 3:**
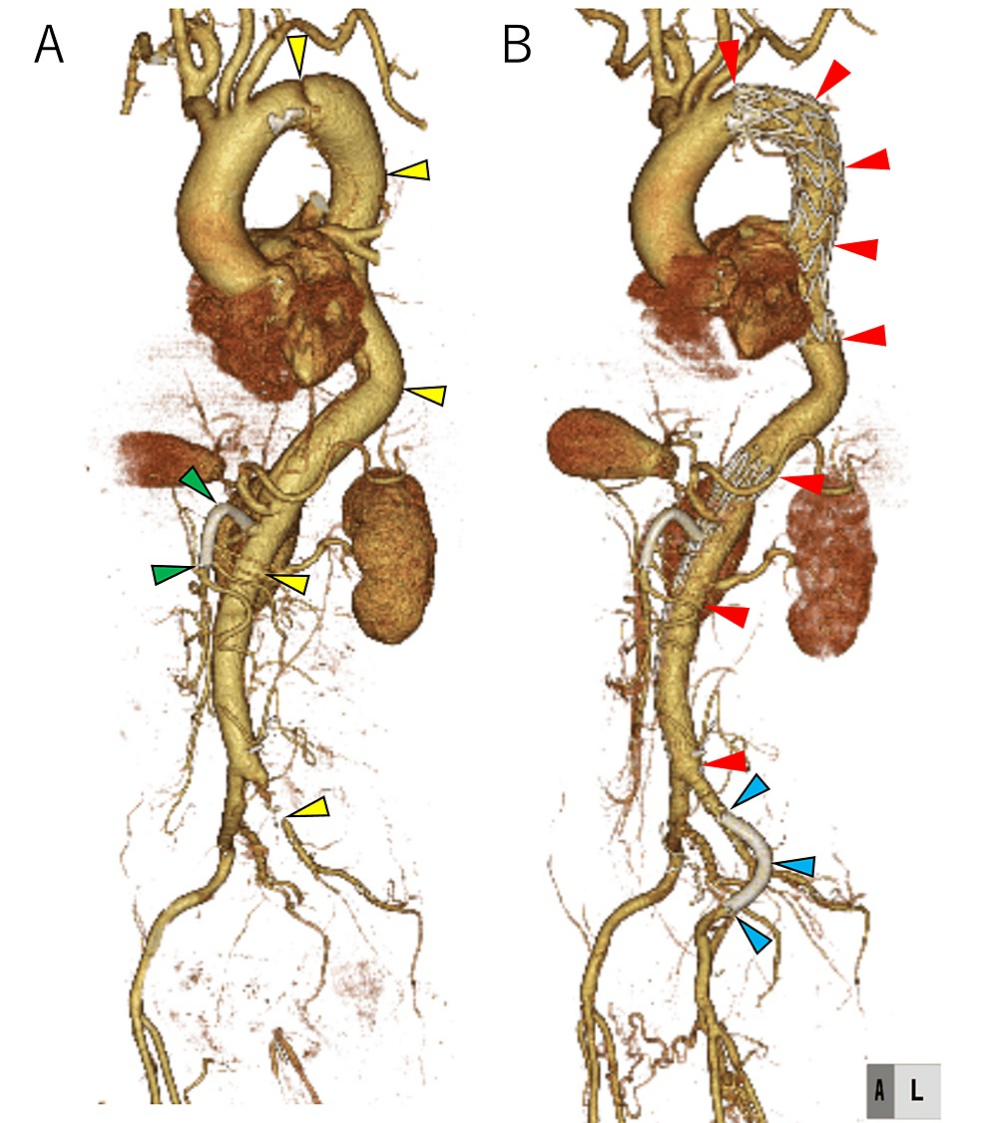
CT image before and after TEVAR Contrast-enhanced CT before and after TEVAR. (A) Before TEVAR, a grade II aortic injury from the left subclavian artery to the terminal aorta was observed (indicated by yellow arrowheads). The stent was placed in the SMA (indicated by green arrowheads). (B) After TEVAR, the stent graft was placed posterior to the left subclavian artery for central entry closure, and the second stent graft was placed for re-entry closure at the terminal aorta (indicated by red arrowheads). The third stent was placed in the left external iliac artery (indicated by blue arrowheads) for residual luminal narrowing. SMA, superior mesenteric artery; CT, computed tomography; TEVAR, thoracic endovascular aortic repair

After the patient’s general condition was stabilized, he was transferred to a nearby hospital. At the transfer hospital, emergency TEVAR using a petticoat procedure and central entry tear closure with posterior left subclavian artery stent placement were done. Additionally, stents were placed posterior to the renal artery for re-entry tear closure and in the narrow left external iliac artery. The final angiography confirmed the absence of adverse events (Figure 3).

Aspirin (100 mg/day) was administered after the SMA stenting. On the third day, the patient gained consciousness and presented without paralysis of the lower extremities.

Subsequently, the postoperative course was marked by persistent diarrhea due to the initial intra-abdominal organ ischemia. In the second postoperative week, edoxaban was started because of the development of deep vein thrombosis (DVT) in the left lower extremity. However, antithrombotic medication was immediately discontinued because of the onset of lower bleeding in an ulcer formed proximal to the sigmoid colon anastomosis, where previous rectal cancer was operated on.

After a few days, the lower bleeding was relieved by stopping the antithrombotics, and 15 mg of edoxaban was administered for DVT.

The patient was transferred to the convalescent hospital on day 46. After one year and four months, the patient was discharged home and has achieved the same life expectancy as before admission.

## Discussion

TEVAR is a less invasive treatment for BTAI and should be chosen, if possible, even in cases of malperfusion. We were unable to perform immediate TEVAR in this case, and open surgery for aortic repair was unsuitable owing to risk factors. Although the introduction of extracorporeal membrane oxygenation could be an option for temporary improvement of ischemia, it was not administered in this case because of the risk of rupture [4]. We decided that the optimum strategy to save the patient's life was to first improve organ ischemia, followed by exploratory laparotomy, and finally TEVAR. In this case, the failure of intraperitoneal organ revascularization by SAC proved a major challenge; however, SAC remained the only treatment option available. The concern was difficulty in performing SAC due to thrombosis of the false lumen, which could be addressed by balloon dilatation. Therefore, SAC was performed as a bridge to TEVAR to restore intraperitoneal organ perfusion. Although there have been reports on SMA stenting for endogenous acute Stanford type B aortic dissection, there have been no reports on SMA stenting for traumatic aortic injury. To the best of our knowledge, this is the first report of this procedure [5]. The time course from ischemia to reperfusion was four hours for the SMA and five hours for the CA.

Subsequently, catheterization of the CA may be unnecessary if collateral blood flow from the SMA to the CA is confirmed [6,7,8]. The longer the time required for catheterization, the more invasive it is to the patient and the longer the time required for definitive care. However, due to residual aortic dissection, maintaining sufficient collateral flow with high blood pressure was challenging; we, therefore, aggressively pursued CA treatment. 

As this case shows, the main focus of BTAI treatment was to stabilize the patient’s general condition before TEVAR, even in the presence of perfusion insufficiency.

## Conclusions

The treatment of BTAI with ischemia is challenging and treatment strategies are limited. In this setting, SAC may be an option as a bridge to TEVAR. In addition, rapid TEVAR may be desirable in BTAI with ischemia; however, stabilization of the patient's general condition should be a priority.
